# Evaluating the use of zein in structuring plant-based products

**DOI:** 10.1016/j.crfs.2020.03.004

**Published:** 2020-03-21

**Authors:** Kristin D. Mattice, Alejandro G. Marangoni

**Affiliations:** Department of Food Science, University of Guelph, 50 Stone Road E, Guelph, Ontario, N1G 2W1, Canada

**Keywords:** Meat analogues, Plant-based, Cheese, Chicken, Gluten

## Abstract

The recent interest in plant-based foods has brought upon the need to develop novel structures using plant-based proteins. However, there is still room for improvement in the development of plant-based meat and cheese alternatives. The rheological properties of self-assembled zein networks were examined to evaluate potential in animal protein replacement. These plant-based protein networks were compared to gluten networks (a common ingredient in current plant-based products), chicken muscle tissue, and cheddar cheese. All samples were analyzed using temperature, amplitude, and frequency sweeps at different time points. Zein networks exhibited unique viscous behaviour (in line with that of an entangled polymer solution), in each amplitude, frequency and temperature sweeps, however only when freshly formed. The results suggest that the bonds and interactions responsible for strengthening zein networks need at least 24 h to fully form. Analysis of the secondary structure by FTIR revealed that zein undergoes a structural reorganization from intermolecular to intramolecular β-sheets during this time, but the substantial content of α-helix structures remains unchanged. Overall, different aspects of zein network rheological behaviour can be compared to either chicken breast, or cheddar cheese, presenting opportunities for zein in plant-based food structuring.

## Highlights

- The rheological behavior of zein networks is investigated
- Rheological properties of zein networks stabilized after 24 h of storage
- Zein networks and cheddar cheese are brittle and demonstrate melting behavior
- Zein networks and chicken muscle tissue demonstrate similar elastic properties

## Graphical abstract

**Figure:**
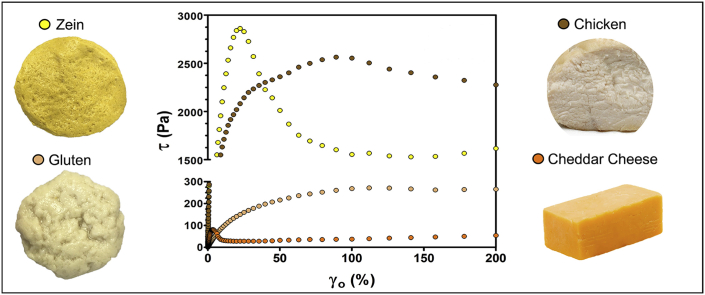

## 1 Introduction

Plant-based proteins can provide the necessary protein quantity and quality in the diet. Consuming an increasingly plant-based diet also provides significant environmental benefits, especially when compared to a diet rich in animal products (Hallström et al., 2015). As a result, the prevalence of veganism and vegetarianism, and the demand for plant-based products, is continually increasing. However, the functionality of different plant-based proteins can differ significantly from each other, and are generally unable to match the functionality of animal proteins without extensive processing (Dekkers et al., 2018). The creation of innovative plant-based products, including meat analogues, can therefore be reliant on an in depth understanding of individual protein functionality.

While animal products contain more than protein, the protein component is responsible for creating characteristic structure and physical properties in many cases. However, developers of many plant-based animal alternatives have had to look to non-protein ingredients to provide the required functionality. Many plant-based cheese products make use of starches and gums to provide texture and melt-stretch properties. Additionally, meat analogue products are often limited to ground meat applications due to the challenges in mimicking the complex structure of muscle tissue, and starches are frequently added to bind the product together (Malav et al., 2015). The proteins that are most commonly utilized in plant-based products are sourced from soy, pea and wheat. However, the desirable functionality achieved with these proteins typically results from texturizing or extrusion processes. This processing adds significant cost to the final products, requiring expensive investment for equipment, and a high energy input (Liang et al., 2002).

It is therefore desirable to identify plant proteins with natural functionality that is more suited to act as animal protein replacements. One protein of particular interest is zein, from maize. Zein is composed of α, β, γ and δ fractions, though, the process of producing commercial zein results in material that is composed primarily of the α fraction. With a high proportion of non-polar amino acids, zein is characteristically water insoluble. The high hydrophobicity of this protein causes zein to self-associate into viscoelastic networks within aqueous environments (Argos et al., 1982). When zein is hydrated and heated above its glass transition temperature (T_g_), it forms a flexible, bendable mass which may be pulled, stretched and sculpted, increasing the opportunity to create structures that can be tailored to the application. Because zein readily solubilizes in aqueous-alcohol solvents, the majority of structural and rheological characterization has been performed in these environments. Therefore, an in depth characterization of zein's physical properties in aqueous environments is required to uncover zein's potential in the area of food structuring and animal protein replacement.

Zein is readily available as a by-product of corn starch and corn syrup production for both food and bioethanol (Anderson and Lamsal, 2011). It is considered GRAS (FDA, 2018), with food applications including water resistant films and encapsulation of bioactives within nanoparticles (Chuacharoen and Sabliov, 2016, Corradini et al., 2014, Shukla and Cheryan, 2001, Zhong and Jin, 2009). From a nutritional standpoint, protein quality can be quantified using the Protein Digestibility-Corrected Amino Acid Score (PDCAAS), which considers both amino acid requirements and digestibility (Schaafsma, 2000, Schaafsma, 2012). Maize has a score of approximately 0.47, and while this is significantly lower than the values of animal protein sources (close to or equal to 1.0), it is comparable to the scores of wheat (~0.44) (Boye et al., 2012, Singh et al., 2008, Taylor and Taylor, 2018). This therefore means that zein could be treated similarly to plant-based products containing wheat proteins, where complimentary proteins are added to improve the nutritional value of the product while the functionality is not lost. The price point of zein is currently higher than other plant-based proteins, though this mainly has to do with the limited yearly production and lack of widespread utilization (Liu et al., 2019, Shukla and Cheryan, 2001). Therefore, the cost of zein could improve given a greater interest in the protein.

Zein shares many characteristics with wheat gluten, which has become an exceptionally common ingredient in plant-based products. In fact, many of the meat analogue products that are considered to have the most meat-like characteristics contain gluten. Both zein and gluten are prolamins that are water insoluble and will self-assemble into flexible, viscoelastic masses when hydrated and heated above their respective glass transition temperatures (T_g_). Although, gluten undergoes a glass transition well below ambient temperatures when hydrated, making it pliable and easy to work with under normal conditions (Almutawah et al., 2007, Attenburrow et al., 1990, Delcour et al., 2012). On the surface, this suggests great potential for the use of zein specifically in meat analogue applications. However, wheat gluten network development and strength results from the formation of disulfide bonds between the cysteine residues of glutenins and gliadins, the two major fractions of gluten (Bache and Donald, 1998, Tuhumury et al., 2014). Hydrogen bonds and hydrophobic interactions between the protein fractions simply aid in strengthening the network further (Belton, 1999, Ng and McKinley, 2008). In contrast, primarily non-covalent interactions are responsible for network formation in zein (Smith et al., 2014). A contributing factor to this difference is the fact that commercial zein contains primarily α-zeins, and this fraction only contains one to two cysteine residues per subunit (Shewry and Halford, 2002). The different bonds involved in network formation give rise to differences in the structure and rheological properties between zein and gluten networks, however it is unclear whether the presence of covalent bonds is critical to gluten's success in animal protein replacement.

In studies of plant-protein functionality for the purpose of developing animal alternatives, zein is repeatedly overlooked. The focus of this work was to study self-assembled zein networks from a fundamental structural and rheological standpoint. This evaluation was performed on zein networks separate from complex food matrices in order to attribute the observed properties to the proteins with confidence. The complimentary analysis of wheat gluten allowed for direct comparison with a similar protein that is currently extensively used in plant-based foods. To fully realize the potential of zein in animal protein replacement, the samples of commercially available cheddar cheese and pre-cooked chicken strips were also compared.

## 2 Materials and methods

### 2.1 Materials

Maize zein was obtained from Flo Chemical Corp (Ashburnham, MA). Medium cheddar cheese, pre-cooked chicken strips and gluten flour (100% vital wheat gluten) were purchased from local supermarkets.

### 2.2 Differential scanning calorimetry

A Mettler Toledo differential scanning calorimeter (DSC) (Mettler Toledo, Mississauga, ON, Canada) was used to determine the glass transition temperature (T_g_) of zein. The effect of water plasticization, or decrease in T_g_, with increasing water activity (a_w_) was observed by equilibrating powdered zein in sealed containers at 25 °C over different saturated salt solutions for three weeks: KNO_3_ (a_w_ = 0.93), KCl (a_w_ = 0.84), NaCl (a_w_ = 0.75), Ca(NO^¬3^)_2_ (a_w_ = 0.51), MgCl_2_ (a_w_ = 0.33), and LiCl (a_w_ = 0.25). Equilibrated protein samples were weighed into aluminum crucibles (6–8 mg) and subjected to the following conditions: 5 °C for 10 min; 5 °C–120 °C at 5 °C/min; 120 °C for 10 min; 120 to 5 °C at 10 °C/min; 5 °C for 10 min; 5 °C–120 °C at 5 °C/min. The T_g_ was determined using STARe software (Mettler Toledo) and the exact temperature was taken at the inflection point of the reversing heat flow signal. After equilibration, the exact a_w_ of the zein was analyzed using an AquaLab water activity meter (Decagon Devices, Pullman, USA). Equilibration was performed in triplicate and each repetition was included as a separate point in the plot of a_w_ against T_g_.

### 2.3 Proximate analysis

Proximate analysis was carried out for zein and gluten. Ash, moisture, fat and protein content were each determined in triplicate. Carbohydrate content was determined by subtraction, with the total of all components adding to 100%. Ash content was determined through dry ashing of samples in a furnace at 550 °C for 5–8 h. Moisture content was determined by drying samples in a vacuum oven held at 70 °C and 30 mmHg for 3–5 h. The soxhlet method was carried out for fat extraction, where 3–4 g of zein was weighed into dry extraction thimbles. The thimbles were then placed in the soxhlet extraction apparatus. Approximately 200 mL of petroleum ether was poured into the flask and was heated gently to allow a continual reflux of petroleum ether for 4–5 h. The sample was removed and the majority of petroleum ether was then poured off from the extraction chamber. The remaining petroleum ether was evaporated in a drying oven set to 60 °C. Protein content was determined using the Dumas combustion method in a LECO FP-528 Protein/Nitrogen Analyzer (Leco Corporation, St. Joseph, MI, USA). The conversion factor used to calculate protein content was 6.25 for zein, and 5.70 for gluten flour (Esen, 1980, Jones, 1931). The composition of pre-cooked chicken strips and cheddar cheese was obtained from the nutritional information on the package. While there are limitations associated with using this provided nutritional information, this work only required the ability to identify the general composition, for which this information was sufficient. The moisture content of these products was calculated by subtraction.

### 2.4 Protein network sample preparation

Particulate zein or gluten flour was weighed into beakers and water was added in excess (at least 10:1 by weight). The proteins were stirred to thoroughly disperse within the water, and were put in an incubator at 40 °C for at least 30 min to allow network formation. Excess water present after network formation was discarded. Zein samples were stored at 40 °C until analysis at different time points: freshly prepared that day (0 h), 24 h (24 h), 48 h (48 h) and one week (1w). Gluten network samples were stored at 40 °C and analyzed after 30 min.

### 2.5 Small amplitude oscillatory dynamic rheology

Shear oscillatory experiments were performed with a rotational rheometer (MCR 302, Anton Paar, Graz, Austria) fixed with parallel plate geometries (20 mm diameter). The geometries were fixed with adhesive 600 grit sandpaper to minimize slip during measurements. Samples were placed onto the lower plate and compressed between the plates with an axial force not exceeding 2N to avoid error, as overloading has been shown to modify the rheology (Macias-Rodriguez and Marangoni, 2016a). The parallel plates had temperature control by Peltier units located in the lower plate and the hood of the rheometer. The exposed edges of the samples were coated with mineral oil to prevent drying or hardening during testing. RheoCompass software and firmware (Anton Paar, Graz, Austria) provided the storage modulus (G′), loss modulus (G″), and shear stress (τ) values used for analysis.

Amplitude sweep tests were performed with shear strain increasing from γ = 0.001% to γ = 300%. The angular frequency (ω) was constant at 3 rad/s. For all samples, the temperature (T) was constant at 50 °C. Frequency sweeps were performed with ω increasing from 0.1 to 60 rad/s. For all samples, γ was constant at 0.01% rad/s and T was constant at 50 °C. Finally, temperature sweeps were performed with T increasing from 5 to 100 °C at a rate of approximately 5 °C per minute. The shear strain and angular frequency were constant at γ = 0.01% and ω = 3 rad/s, respectively. Samples were analyzed in triplicate and plotted with outliers removed.

### 2.6 Attenuated total reflectance - fourier transform infrared spectroscopy

Samples were compressed to thoroughly remove any unbound water prior to analysis. Infrared spectra of the different zein masses were obtained using an Fourier transform infrared spectrometer (FTIR) (model IRPrestige-21, Shimadzu Corp., Kyoto Japan). The FTIR was equipped with an attenuated total reflectance (ATR) accessory (Pike Technologies, Madison, WI, USA). Samples were scanned from 600 to 4000 cm^−1^ with a 4 resolution and 32 scans per spectrum. Analysis was carried out in triplicate at ambient temperatures. Secondary structure analysis of zein fibres was conducted by deconvoluting the amide I region (1600-1700 cm^−1^) using OriginPro software (Origin Lab Corp., Northampton, MA, USA). The second derivative of the original spectra was used to locate each of the underlying bands, and the wavenumber position was fixed during fitting. Each band was fitted using the Lorentzian function. The secondary structural content was determined from the relative areas of the individual bands in the amide I region of zein (Li et al., 2009, Mejia et al., 2007, Moomand and Lim, 2015, Zhang et al., 2011).

### 2.7 Statistical analysis

GraphPad Prism 5.0 (GraphPad Software, San Diego, CA, USA) was used for statistical analysis and logarithmic transformation of data. A two-tailed unpaired *t*-test was performed on the secondary structure data by FTIR for each sample type. The confidence level was chosen as 95%. One-way ANOVA was performed to determine significance between the slopes of log(G′) – log(ω) plots obtained from frequency sweeps. The level of significance was chosen as *p* < 0.05.

## 3 Results and discussion

### 3.1 Proximate analysis

Zein and gluten were analyzed to determine the crude content of protein, fat, moisture and ash (Table 1). The content of carbohydrates was determined by subtraction. The composition of pre-cooked chicken strips and cheddar cheese (calculated based on package nutritional labelling) was included for comparison. The obtained results confirm that protein is the major component in both the powdered zein and gluten. However, some differences existed between the proteins, where zein had greater proportions of protein, ash and lipids, while wheat gluten had greater proportions of moisture and carbohydrates. The obtained pre-cooked chicken strips and cheddar cheese also contained a significant amount of protein, however there were also large quantities of fat and moisture present due to the fact that these samples were whole food matrices. In particular, cheddar cheese had a greater content of fat than protein, which must be accounted for during further analysis.

**Table 1:** Proximate analysis of commercially available gluten flour and powdered zein, compared with the composition of commercial products calculated from the package nutrition label. Values are presented as averages ±standard deviation.

	Gluten Flour	Zein	Pre-Cooked Chicken	Cheddar Cheese
Moisture	6.7% ± 0.09	3.83% ± 0.15	75.2%	42.1%
Ash	0.71% ± 0.02	1.70% ± 0.005	0.4%	0.7%
Fat	0.56% ± 1.0 × 10^−4^	2.87% ± 0.003	2.1%	33.3%
Protein	71.0% ± 0.11	87.20% ± 0.49	22.3%	23.8%
Carbohydrates	~21.2%	~4.4%	0%	0%

### 3.2 Glass transition temperature

The T_g_ of zein at different water activities was observed by subjecting proteins to two heating cycles, where the proteins were heated to 120 °C in each. A peak associated with native zein protein was observed during the first heating cycle in all samples, occurring at approximately 60–65 °C. This peak ultimately overlapped with the T_g_ in certain samples, rendering it impossible to distinguish the location of the glass transition. Therefore, the T_g_ was recorded during the second heating cycle for all samples.

**Fig. 1:**
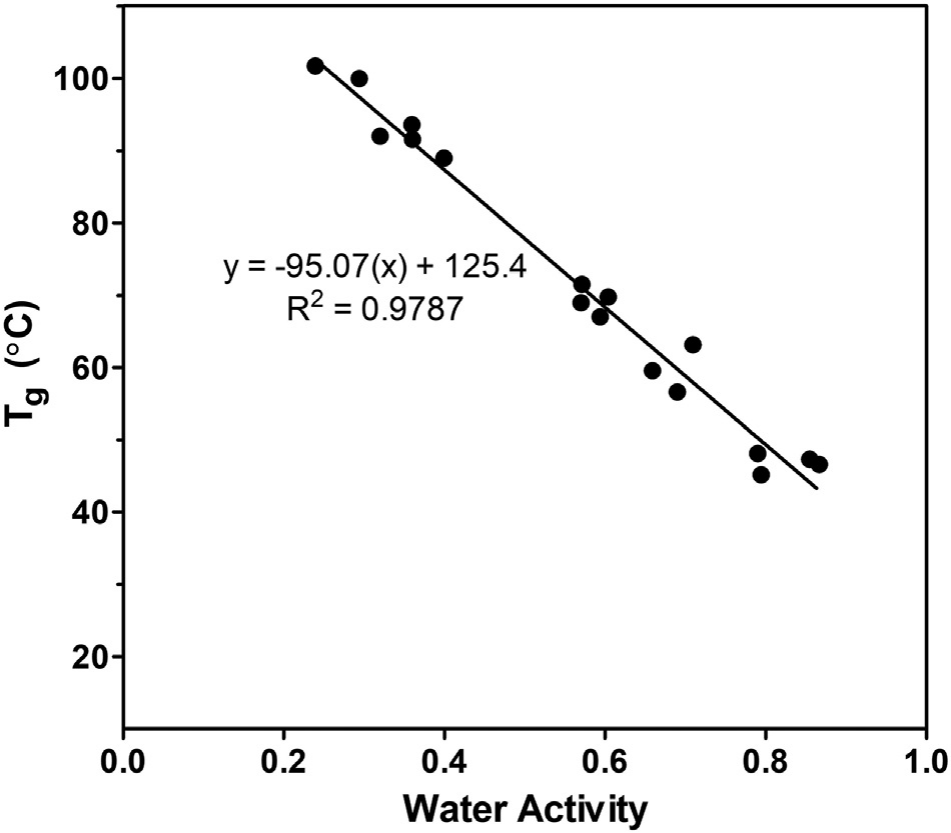
Plot of water activity against the measured glass transition temperature (T_g_) of zein.

As has been shown previously in various studies (Erickson et al., 2014, Lawton, 2004), the presence of water causes a pronounced decrease in zein's T_g_ (Fig. 1). The observed decrease had a linear relationship with increasing a_w_ of the sample (p < 0.05; R^2^ = 0.9787). The lowest obtained T_g_ was approximately 45 °C, identified when the a_w_ was greater than 0.8. Due to this result, further experimentation was carried out at temperatures equal to or greater than 50 °C, in an excess of water, to ensure zein would exist in its rubbery state.

### 3.3 Amplitude sweep

The storage and loss moduli (G′ and G″) of zein networks were obtained at different time points (0 h, 24 h, 48 h and 1w of storage) over increasing shear strain (γ = 0.001%–300%). This type of small amplitude rheological analysis probes the linear viscoelastic region (LVR) and can elucidate differences in material functionality. The LVR is characterized as the region of viscoelasticity where the material produces constant values of storage and loss modulus within a specific range of applied strain (Kasapis and Bannikova, 2017, Ortolan et al., 2017).

**Fig. 2:**
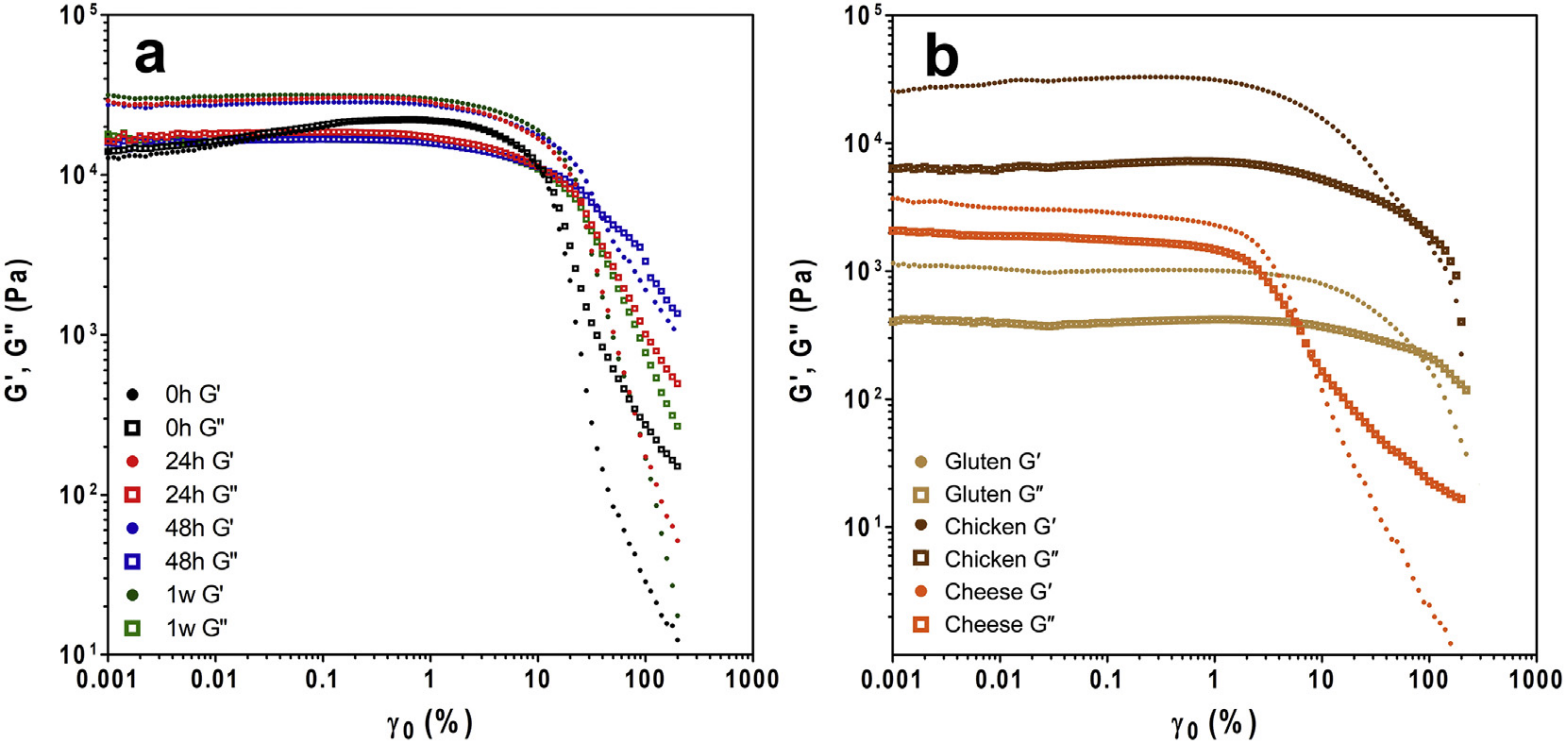
Amplitude sweeps of a) zein at different storage time points; and b) gluten network, pre-cooked chicken strips and cheddar cheese (T = 50 °C; ω = 3 rad/s).

Zein samples exhibited linear viscoelasticity at low to mid strain rates throughout one week of storage (Fig. 2). At most time points, G′ values were greater than G″ in the LVR, indicating the existence of solid, gel-like characteristics. The exception to this trend were the 0 h samples (Fig. 2a) where G″ values remained slightly above G′ values throughout the applied strain rates until the material yielded. This indicated that freshly formed zein networks demonstrated greater viscous properties, contrasting samples stored for 24 h or more. These viscous properties were also observed by Madeka and Kokini (1996), who described the behaviour as entangled polymer flow (Madeka and Kokini, 1996). Storage for at least 24 h increased the G′ values above the relatively unchanging G″ values in the LVR, such that the networks did not exhibit reduced viscous properties, but rather significantly increased elastic properties. A similar observation was made in a recent study, where zein in aqueous ethanol appeared to form more dense and organized structures with age, determined using ultra-small angle x-ray scattering (Uzun et al., 2017).

Another difference in behaviour was observed at the point where non-linear behaviour begins. This is characterized by the yield stress, which is defined here as the point where G′ and G″ crossover (tan δ = 1). Due to the unique viscous behaviour that zein initially demonstrates (at 0 h), no crossover point was identified until reaching the 24 h time point. Still, a sharp decrease of G′ and G″ values at a higher strain rate (approximately 10%) is visually apparent in 0 h samples (Fig. 2a). The yield stress increased after reaching at least the 24 h storage time point, occurring at approximately 25% but not increasing further with additional storage time. This suggests that while it takes 24 h for zein networks to fully form, there is no time dependence on network and bond formation beyond this.

The behavioural similarities between gluten networks and chicken, particularly in high yield stress values, explain its extensive use in structuring meat analogues at present. However, the values of G′ and G″ were significantly lower than that of chicken muscle tissues in each case, indicative of a notable textural difference between chicken muscle tissue and gluten based meat analogues. There is therefore potential for improvement with the use zein, as the elastic characteristics of aged zein samples were similar to that of chicken (Fig. 2b). However, efforts to increase the yield stress are required as zein networks yielded at lower strain rates than both chicken (~γ = 80%) and gluten (~γ = 70%) samples. From a different side, the yield stress of zein was more similar to that of cheese, which occurred at strain rate of approximately 7%. These results point to some potential for the use of zein in plant-based cheese applications. However, the G′ and G″ values of zein samples were significantly greater than those obtained for cheddar cheese, pointing to the need for plasticization to increase feasibility.

**Fig. 3:**
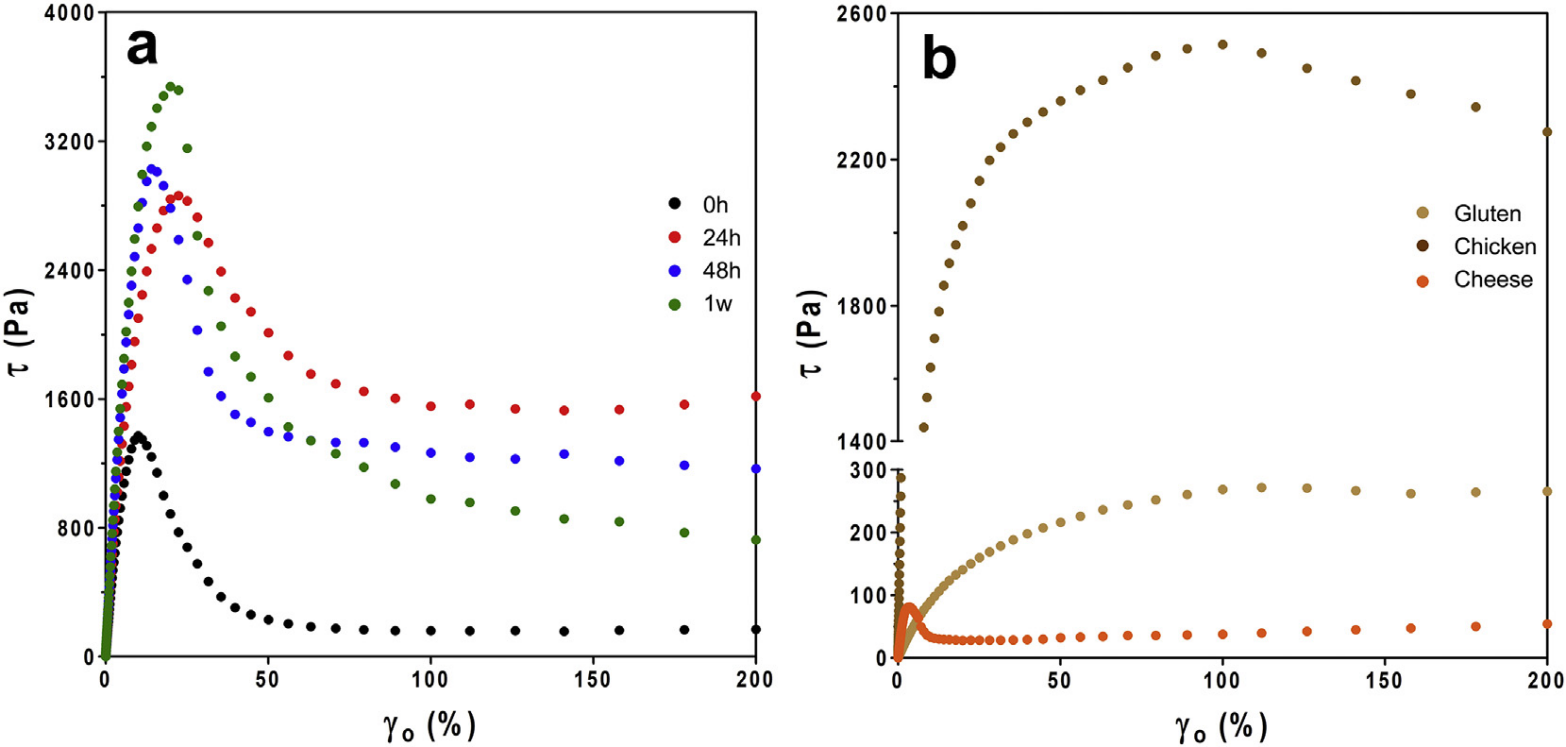
Stress versus strain plots of a) zein at different storage time points; and b) gluten network, pre-cooked chicken strips and cheddar cheese (T = 50 °C; ω = 3 rad/s).

The shear stress values (τ) were also obtained simultaneously during amplitude sweeps at T = 50 °C and have been plotted against shear strain (Fig. 3). These stress-strain curves highlight the brittle behaviour of zein networks, where very little applied strain compromises the material (Fig. 3b) (Macias-Rodriguez and Marangoni, 2018). The term brittle is used here to describe that the material's structure is irreversibly damaged or broken after reaching a maximum stress value. Notably, the peak maximum occurred at approximately the same shear strain value, regardless of storage time, though the maximum shear stress reached for each sample increases with increasing storage. The behaviour of gluten networks and chicken contrasted this greatly, as stronger, more ductile materials (Fig. 3a). This result suggests that ductile deformation could be a critical physical attribute for muscle tissue and meat analogues, and that the brittle nature of zein compromises its ability to fulfill the functional requirements as an ingredient in meat analogue applications. However it was promising that higher shear stress values were observed, closer to that of chicken. Gluten and cheese samples demonstrated significantly lower values, indicative of much softer materials. Together, these results suggest that determining methods to increase the yield strength of zein networks are required to produce materials statistically similar to cooked chicken muscle tissue. On the other hand, the brittle characteristics do not appear to be disadvantageous when looking at the similarities between zein and cheese samples. When considering zein in the application of plant-based cheeses, it is instead the hardness of the zein networks that reduces practicality. Therefore, it is also desirable to determine methods to decrease the hardness of zein networks to produce materials statistically similar to cheese products.

### 3.4 Frequency sweep

The dependence of the samples on frequency was analyzed at γ = 0.01%, which was determined to be within the LVR for all samples. Frequency sweeps provide information about changes in the viscoelastic properties of the polymer network, including the structure and the interactions between the polymers. Typically, a greater frequency dependence is observed in more fluid-like materials, while gels characteristically have little to no frequency dependence (Gabriele et al., 2001, Kasapis and Bannikova, 2017, Macias-Rodriguez and Marangoni, 2016b, Ortolan et al., 2017, Steffe, 1996).

**Fig. 4:**
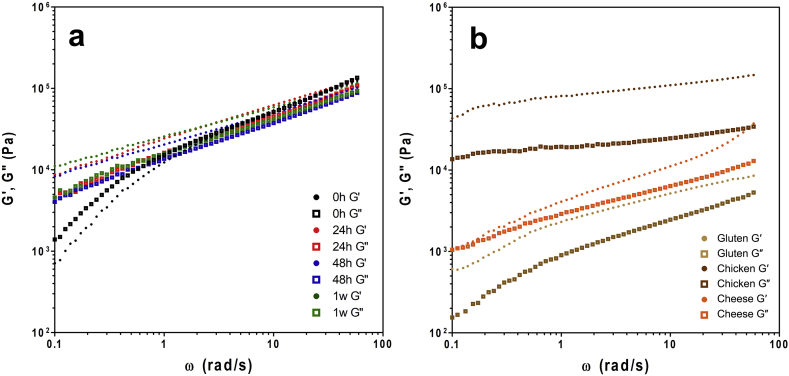
Frequency sweeps of a) zein at different storage time points; and b) gluten network, pre-cooked chicken strips and cheddar cheese (T = 50 °C; γ = 0.01%).

Frequency sweeps performed at 50 °C (Fig. 4) revealed that there was a weak frequency dependence for zein networks at all time points. Accordingly, the slopes of the linear regions of each log(G′) – log(ω) plot (Table 2) were each less than 1, behaviour matching that of a weak gel. Though, freshly formed zein networks had greater frequency dependence than zein samples at all other time points. This coincides with the fact that these 0 h samples were uniquely viscous, with G″ > G′ throughout. The two values also approached each other as ω increased, behaviour reminiscent of a concentrated or entangled solution, where no covalent bonds exist to link a network (Kasapis and Bannikova, 2017, Steffe, 1996). Zein networks then developed the behaviour of a weak gel after the 24 h time point, and differences between time points after this were almost indistinguishable.

**Table 2:** Slopes from log(G′)–log(ω) and log(G″)–log(ω) plots (data obtained from frequency sweeps samples at 50 °C and γ = 0.01%). Values with the same superscript letter are not significantly different (p < 0.05).

Zein Sample		Slope	R^2^	Comparative Sample		Slope	R^2^
0 h	G′	0.76 ± 0.021^a^	0.956	Gluten Network	G′	0.41 ± 0.011^cf^	0.965
	G″	0.65 ± 0.016^b^	0.968		G″	0.51 ± 0.011^e^	0.976
24 h	G′	0.42 ± 0.001^cd^	0.999	Chicken Strips	G′	0.16 ± 0.003^i^	0.976
	G″	0.48 ± 0.003^eg^	0.997		G″	0.12 ± 0.003^i^	0.966
48 h	G′	0.39 ± 0.001^cf^	0.999	Cheddar Cheese	G′	0.50 ± 0.005^e^	0.994
	G″	0.46 ± 0.002^deg^	0.999		G″	0.38 ± 0.003^f^	0.996
1w	G′	0.37 ± 0.001^f^	0.999				
	G″	0.44 ± 0.003^cg^	0.998				

There was a greater magnitude of difference between the G′ and G″ values of gluten networks and chicken than for zein networks. This results points to the fact that both of these materials were more strongly bound together than zein networks. Chicken samples displayed very low frequency dependence with the lowest slope of all samples, characteristic of highlight structured, and solid materials. In contrast, cheese samples displayed greater frequency dependence (Table 2), which is logical given that cheeses exist in a partially melted state at 50 °C, primarily due to the melting of milk fat. Once again, there can be two perspectives drawn from looking at this data. First, the magnitudes of G′ and G″ values were the greatest for zein and chicken samples (Fig. 4), giving zein an advantage over gluten as ingredients in meat analogues. Although, the results emphasize the fact zein networks are not as strongly bound together as chicken muscle tissue and gluten networks. This could reflect the strength of the non-covalent bonds within the network, but is likely more indicative of the established absence of covalent bonds within zein networks. This again points to the need to strengthen zein networks in order to obtain behaviour more similar to that of muscle tissue. If covalent bonds are indeed the determinant factor, future research should investigate the use of crosslinking techniques for zein (Glusac et al., 2018, Masamba et al., 2016, Reddy et al., 2009). The second perspective looks at the similarities between the frequency dependence of zein networks and cheese samples. With yet another behavioural similarity between zein and the cheese samples, the prospective of plant-based zein cheeses is highlighted, but raises the issue of the substantially greater hardness of zein networks. Although, plasticizing zein has been accomplished previously using food grade ingredients (Chaunier et al., 2017, Erickson et al., 2014, Ghanbarzadeh et al., 2006) and therefore will likely not be a daunting task. In addition, the eventual plant-based cheese products produced would include other non-protein ingredients in a food matrix, and as such, the hardness of zein may not be undetectable once the contributions of other ingredients are accounted for.

### 3.5 Temperature sweep

**Fig. 5:**
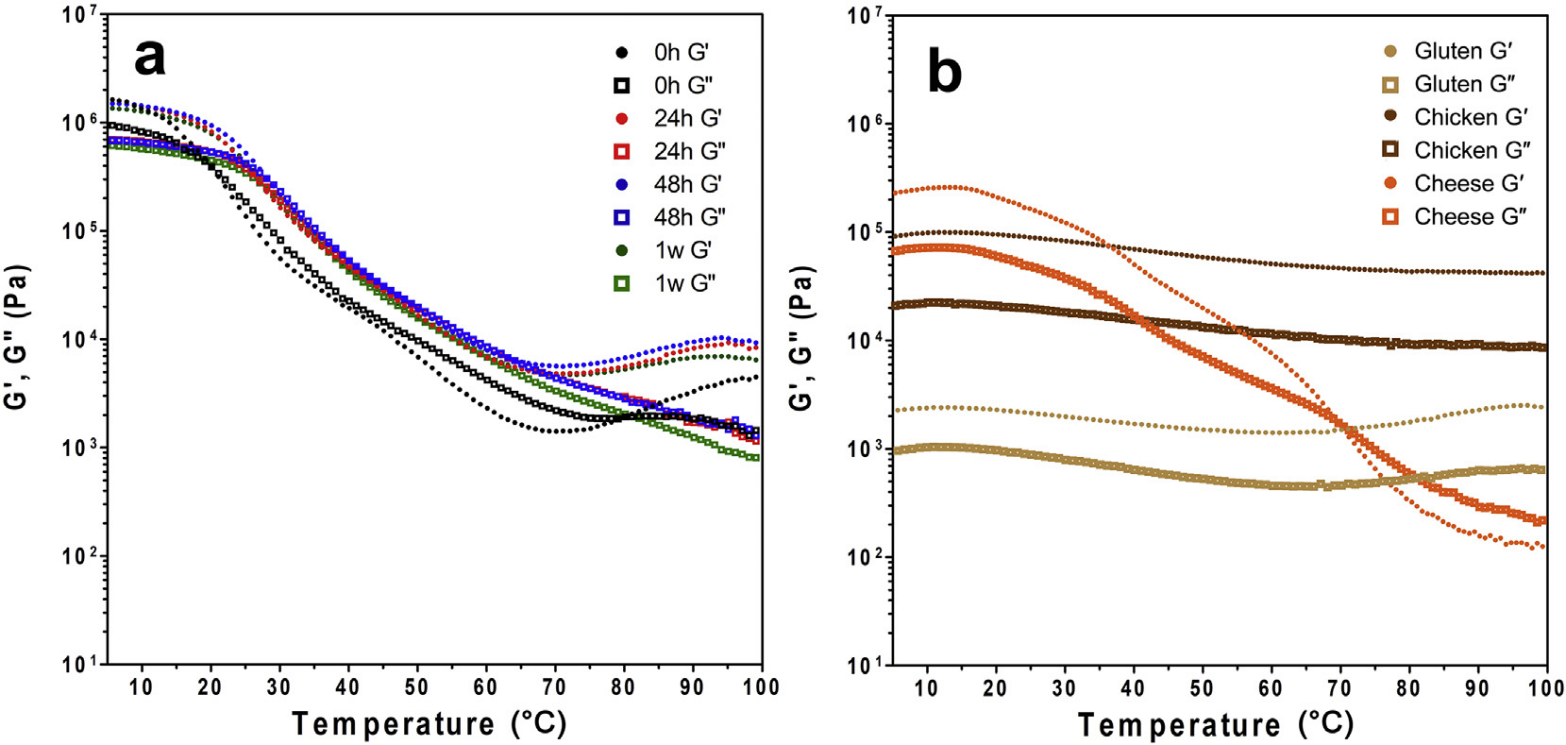
Temperature sweeps of a) zein at different storage time points; and b) gluten network, pre-cooked chicken strips and cheddar cheese (γ = 0.01%; ω = 3 rad/s).

Temperature sweeps are used to evaluate thermo-mechanical structural modifications and determine the temperatures at which these changes occur. Zein networks at all storage time points initially softened with increasing temperature (Fig. 5a), likely consequent of the weakening of non-covalent bonds at higher temperatures (Gabriele et al., 2001). This initial decrease was the steepest for 0 h samples, likely resultant of the weak structure formed at that time. Above 50 °C, the G′ fell once again, until a crossover occurred at approximately 70 °C (80 °C for 0 h samples), when the material became more elastic. There was a point in the range of 45 °C where G′ began to approach G″ briefly, most easily seen in 0 h samples. Since zein's T_g_ was observed at approximately this temperature, it is likely that the conversion from glassy to rubbery domains causes a brief increase in the elastic modulus in the material. Similar to the findings from the amplitude and frequency sweeps, zein did exhibit a more elastic response after reaching the 24 h time point. However, all zein networks demonstrated a greater viscous component in the range of approximately 20–70 °C, where G′ values remained only slightly below G″ values. In addition, the difference between the points of maximum and minimum hardness (G′_max_ and G″_max_ vs. G′_min_ and G″_min_) was extreme, where zein was essentially demonstrating melting behaviour in this range of temperatures.

Gluten networks and chicken remained elastic (with G′ > G″) throughout the temperature range of 5–100 °C, with minimal fluctuation in G′ and G″ values and magnitude of difference (Fig. 5b). As expected, cheese demonstrated melting behaviour over this range of temperatures, with G′ > G″ initially, but crossing over to more viscous properties after approximately 70 °C. While it has been mentioned previously that zein shares a number of rheological characteristics with cheese, this similar softening or melting behaviour with increasing temperature is the most substantial result and highlights the potential of zein in structuring plant-based cheese products. In addition, this functionality was achieved with protein alone, indicative of great potential for plant-based zein cheese products with significantly reduced fat contents. Still, efforts to plasticize and soften zein may be necessary to yield products that more closely mimic the properties of cheeses. While the melting observed is not at all reminiscent of muscle tissue, it is possible that the previously discussed efforts to strengthen zein networks could reduce the softening observed at high temperatures. This includes promoting the formation of covalent bonds within self-assembled zein networks to produce more ductile deformation properties, similar to what is observed with gluten networks and chicken muscle tissue. Previous observations that covalently linked cheese products do not demonstrate significant melting properties provide the basis for this view (Li et al., 2019, Lucey et al., 2003).

### 3.6 Secondary structure

The amide I and II bands, positioned at 1645 cm^−1^ and 1540 cm^−1^ respectively, appear due to the vibration and stretching of amide bonds within the protein structure. The amide I band is predominantly caused by the stretching of the carbonyl (C=O) group, while the amide II band is primarily associated with the N–H bending and CN stretching vibrations. It has been well established that the amide I band is made up of numerous underlying bands, which can be used to determine the presence of different secondary structures (Byler and Susi, 1986). The values used here consist of: intermolecular β-sheets at 1610-1625 cm^−1^, intramolecular β-sheets at 1630-1640 cm^−1^, random coil at 1640-1648 cm^−1^, α-helices at 1648-1658 cm^−1^, β-turns at 1660-1668 cm^−1^ and intramolecular β-sheets at 1670-1684 cm^−1^ (Byler and Susi, 1986, Kong and Yu, 2007, Moomand and Lim, 2015).

**Fig. 6:**
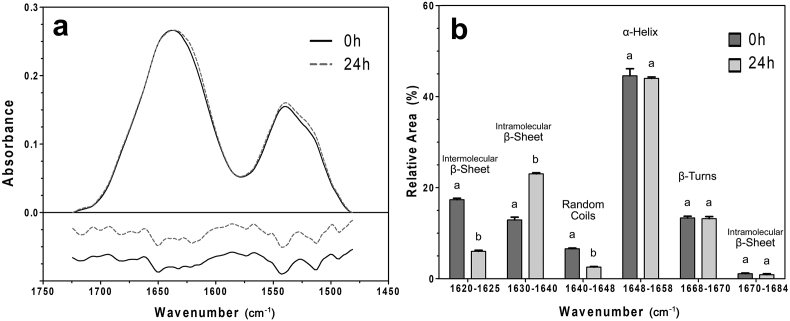
FTIR data presented as a) the spectra of the amide I and II region and second derivative of zein at different time points; and b) relative area of bands deconvoluted from the amide I band (1700-1600 cm^−1^). Values presented are averages (n = 9). Error bars represent the standard deviation. Bars labelled with the same letter are not significantly different.

Since it was determined that the rheological properties of zein change significantly from the 0 h–24 h time points, but do not change significantly with additional time, only 0 h and 24 h samples were analyzed by FTIR. The amide region of the FTIR spectra of these two samples is shown in Fig. 6. Through the deconvolution of the amide I band it was determined that zein networks contain primarily α-helical structures, in agreement with previous investigations (Fig. 6b) (Forato et al., 2003, Momany et al., 2006, Moomand and Lim, 2015). Overall, variation between the amide I spectra was found only in the region of 1640–1620 cm^−1^. This corresponded to differences in the content of intermolecular and intermolecular β-sheets. These results suggested that zein networks initially form a greater extent of intermolecular β-sheets. However, there was a transition that occurred after 24 h where intramolecular β-sheets began to predominate. This is perhaps a result of zein's high hydrophobicity. When water is introduced, zein quickly aggregates into a large mass, likely occurring to minimize the surface area exposed to the aqueous environment. This environment can therefore be considered a driving force for intermolecular interactions due to the increasing protein-protein contact. With time, zein appears to slightly restructure, potentially caused by the preferred formation of non-covalent hydrophobic interactions that strengthen the network. It is therefore conceivable that the rearrangement to intramolecular β-sheets is preferred, and indicative of a stronger, more stable structure. This concept is also supported by the observed decrease in random structures present after 24 h.

## 4 Conclusions

Overall, zein networks demonstrate unique viscous properties when first formed, but transition into stronger, more elastic structures after 24 h of storage. This is reflected in the secondary structure, with some conversion and reorganization taking place from intermolecular to intramolecular β-sheets during this time. The structure then stabilizes at this point, as increasing the storage time does not impact the rheological properties further. The similarity between the G′ and G″ values of zein and chicken muscle tissue lead to the potential application of zein in meat analogues. However, investigations into methods to improve zein network strength and reduce brittleness are required in order increase the feasibility of producing meat analogues from zein. From a different rheological perspective, zein shares melting characteristics with cheddar cheese and this natural functionality could create opportunities in plant-based cheese applications. As a whole, this work highlights the potential and versatility of zein in different animal protein replacement applications.

## Author contributions

Kristin Mattice carried out the experiments, analyzed the data and wrote the paper. Alejandro Marangoni supervised and managed the research, edited the manuscript and submitted it.

## Declaration of Competing Interest

The authors declare there are no conflicts of interest.
